# Radiotherapy plus lenvatinib versus radiotherapy plus sorafenib for hepatocellular carcinoma with portal vein tumor thrombus: a retrospective study

**DOI:** 10.3389/fphar.2024.1458819

**Published:** 2024-10-15

**Authors:** Min Zhang, Zhuangzhuang Zheng, Qiuhui Ding, Jing Su, Ying Xin, Xin Jiang

**Affiliations:** ^1^ Jilin Provincial Key Laboratory of Radiation Oncology and Therapy, The First Hospital of Jilin University and College of Basic Medical Science, Jilin University, Changchun, China; ^2^ Department of Radiation Oncology, the First Hospital of Jilin University, Changchun, China; ^3^ NHC Key Laboratory of Radiobiology, School of Public Health of Jilin University, Changchun, China

**Keywords:** hepatocellular carcinoma, PVTT, transcatheter arterial chemoembolization, radiotherapy, VEGF

## Abstract

**Background and Aims:**

Portal vein tumor thrombus (PVTT) occurs frequently in hepatocellular carcinoma (HCC) patients. However, there is currently no satisfactory treatment. Radiotherapy (RT) and tyrosine kinase inhibitors (TKI) are currently commonly used. However, whether their combined use provides a survival benefit is debatable. This retrospective study compared the efficacy and safety between radiotherapy plus lenvatinib (RT + L) and radiotherapy plus sorafenib (RT + S) in the treatment of hepatocellular carcinoma with portal vein tumor thrombus (PVTT).

**Methods:**

Among patients with PVTT who received RT + L or RT + S between March 2017 and September 2022, the primary endpoints were overall survival (OS) and progression-free survival (PFS). The secondary endpoints were objective response rate (ORR), disease control rate (DCR), and incidence of treatment-related adverse effects. The prognostic factors were also assessed.

**Results:**

The analysis included 152 patients (RT + L: 48; RL: 25; RT + S: 55; S: 24). Compared with the RT + S group, the RT + L group had a longer OS and PFS. Among patients with type I/II PVTT, the median OS times were 19.8 months and 13.5 months (*p* = 0.047) and the median PFS was 12.3 months and 7.3 months (*p* = 0.042), respectively. And the median OS of the patients with type I/II PVTT were 14.4 months and 8.3 months (*p* = 0.030) and the median PFS was 8.3 months and 6.2 months (*p* = 0.026). ORR and DCR in RT + L group (25.0% and 75.0%) were also little higher than those in RT + S group (20.0% and 70.9%), but not statistically significant. In univariate analysis, etiology, Type of PVTT, alpha-fetoprotein (AFP) level, Child–Pugh score, and treatment method influenced OS. Multivariate analysis confirmed that treatment method, etiology, alpha-fetoprotein (AFP) level, and Child–Pugh score were independent prognostic factors for OS. Similar safety profiles were observed in the RT + L and RT + S groups. The most common adverse events were myelosuppression, decreased liver function, fatigue, diarrhea, nausea, and vomiting. Most adverse reactions were grade 1–2.

**Conclusion:**

The side effects of radiotherapy plus lenvatinib were acceptable. Compared to RT + S, RT + L had good efficacy in the treatment of hepatocellular carcinoma with PVTT. Validation is needed in prospective studies with larger sample sizes.

## Introduction

Hepatocellular carcinoma (HCC) is the most common pathological type of primary liver cancer and the third most common cause of cancer-related deaths. Portal vein tumor thrombus (PVTT) is a key indicator affecting the prognosis and clinical stage of HCC, with an incidence of 44%–62% in patients with HCC (Zhang et al., 2015; Yang et al., 2018). According to the National Comprehensive Cancer Network (NCCN) guidelines, HCC with PVTT formation is classified as Barcelona stage C, with a median overall survival (OS) of only 2.7–4.0 months in untreated patients (Zheng et al., 2022). However, a global consensus or guideline for the treatment of PVTT is lacking. In European countries, sorafenib, used as a first-line therapy, only increases the median OS of patients with PVTT to 5.6 months; hence, better treatment modalities are needed (Yu and Kim, 2015). In addition to sorafenib, recent NCCN guidelines have recommended lenvatinib as a first-line drug for advanced HCC patients (Kudo et al., 2018). In addition, experts from China and Southeast Asian countries believe that multidisciplinary treatments including surgery, transcatheter arterial chemoembolization (TACE), radiotherapy (RT), and/or molecularly targeted drugs should be considered to obtain more satisfactory results (Cheng S. et al., 2020). In particular, for patients who are inoperable or carry PVTT, TACE, RT, and targeted therapy are more suitable treatment options (Cheng et al., 2022; Liang et al., 2024; Cheng H. et al., 2020; Benson et al., 2021).

Lenvatinib is a multi-kinase inhibitor that targets vascular endothelial growth factor (VEGF) receptors one to three, fibroblast growth factor (FGF) receptors one to four, platelet-derived growth factor (PDGF) receptor α, RET, and KIT (Johnson et al., 2013). In a randomized phase 3 clinical trial, lenvatinib was not inferior to sorafenib as a first-line treatment for unresectable HCC, with median OS of 13.6 and 12.3 months, respectively. Compared with sorafenib, lenvatinib showed a significant benefit in median progression-free survival (PFS) (7.4 vs 3.7 months; *p* < 0.0001). Lenvatinib also demonstrated a higher objective response rate (ORR) than sorafenib (21.4% vs 9.2%; *p* < 0.0001). However, the study excluded patients with >50% liver involvement and main portal vein invasion (Kudo et al., 2018). Another phase 3 trial that assessed patient health-related quality of life (HRQOL) during lenvatinib treatment for advanced HCC demonstrated that the evidence of HRQOL benefits compared with sorafenib in clinically relevant domains supported the use of lenvatinib to delay functional deterioration in advanced HCC (Vogel et al., 2021). A prospective randomized study evaluating the efficacy of lenvatinib combined with TACE as the first-line treatment for HCC with PVTT showed a significantly higher median time to progression (TTP) in patients in the lenvatinib group (4.7 vs 3.1 months; *p* = 0.029) and ORR (53.1% vs 25.0%, *p* = 0.039) compared to those in the sorafenib group. Subgroup analysis showed that patients with larger tumor, extrahepatic metastases, PVTT type I/II, and higher alpha-fetoprotein (AFP) level appeared to benefit more from TACE plus lenvatinib (Ding et al., 2021).

In addition to systemic therapy, experts in China and some Asian countries have also recommended RT as the treatment of choice for PVTT (Deng et al., 2022). With the development of RT, three-dimensional conformal RT (3D-CRT), intensity-modulated RT (IMRT), and stereotactic body RT (SBRT) were widely used in the treatment of liver cancer (Rim et al., 2018). A randomized clinical trial by Yoon et al. has shown that in patients with PVTT, the median OS in the TACE-RT group (55.0 vs 43.0 weeks; *p* = 0.04) and the median TTP (31.0 vs 11.7 weeks; *p* < 0.001) were significantly higher than those in the sorafenib group (Yoon et al., 2018). In their retrospective study of 154 and 133 patients with PVTT treated with IMRT and SBRT, respectively, Li et al. have reported similar OS, PFS, intrahepatic control (IC), and local control (LC) between the SBRT and IMRT groups (Li et al., 2021). RT alone or in combination can improve the survival rate and quality of life in patients with PVTT (Yu and Park, 2016). However, the efficacy and safety of RT in combination with lenvatinib for HCC with PVTT have not been assessed. Therefore, this study compared the efficacy and safety of RT plus lenvatinib versus RT plus sorafenib in patients with HCC with PVTT.

## Methods

### Study design and patients

We screened 196 patients diagnosed with HCC and PVTT at the First Bethune Hospital of Jilin University between March 2017 and September 2022. Finally, this study enrolled 152 patients who received Lenvatinib (n = 25), RT plus Lenvatinib (n = 48), sorafenib (n = 24) and RT plus sorafenib (n = 55). HCC was confirmed histologically or clinically based on the Chinese Society of Clinical Oncology guidelines. PVTT was diagnosed using magnetic resonance imaging (MRI), abdominal dynamic computed tomography (CT), or ultrasonic examination. The inclusion criteria were: (1) Age ≥18 years, (2) Definite diagnosis of HCC with PVTT, (3) Liver function Child–Pugh score of A or B, (4) Prior TACE, and (5) Eastern Cooperative Oncology Group (ECOG) score ≤2. The exclusion criteria were: (1) History of malignant tumors in organs other than HCC; (2) Liver function score of C; (3) Any contraindications to RT or lenvatinib treatment; and (4) Receiving other treatments, including radiofrequency ablation, immune checkpoint inhibitors, TACE and iodine 125 seed implantation, at the same time during radiotherapy and lenvatinib/sorafenib treatment. This study was approved by the ethics committee of the institution.

### RT

All patients received external beam RT after diagnosis. Gross tumor volume (GTV) was defined as the tumor volume with high density in the arterial phase, and PVTT volume with filling defects in the venous phase. The clinical tumor volume (CTV) was generated by a 5-mm increase in the GTV to cover any potential micrometastases around the tumor. The planning target volume (PTV) is generated by enlarging the margins of the CTV by 5–10 mm to compensate for changes in the internal physiological motion and size, shape, and position of the CTV. Patients receiving IMRT underwent CT scanning in the supine position with the arms raised over the top of the head for RT planning. The total dose to the PTV was 50 Gy with a fractional size of 2.0 Gy, using 6 MV X-rays with a linear accelerator, five times per week. Patients who received SBRT had the same simulated CT scan process and motion management as those who received IMRT. The total dose to the PTV was 40 Gy, with a fractional size of 8.0 Gy and five fractions administered per week.

### Lenvatinib or sorafenib treatment

All patients in this study were treated with Lenvatinib or sorafenib. Lenvatinib or sorafenib was administered before, during, or after RT. The lenvatinib dose was 12 mg (≥60 kg) or 8 mg (<60 kg) orally once daily. Sorafenib was administered orally at a dose of 400 mg twice daily. When grade 3 or 4 adverse events (AEs) occurred, as defined by the National Cancer Institute Common Terminology Criteria for Adverse Events (CTCAE version 5.0), the drug dose was adjusted according to the instructions until the AE was relieved or eliminated. Lenvatinib or sorafenib treatment was discontinued if unacceptable AEs persisted after dose modification.

### Observation endpoints

The primary observation endpoints were OS and PFS. OS was defined as the time from the first treatment to death from any cause. PFS was defined as the time from the first treatment to tumor progression or death due to any cause. The secondary observation endpoints were the ORR, DCR, and safety. In the subgroup analysis, we compared the results of patients receiving RT plus lenvatinib and those receiving RT plus sorafenib with different PVTT types (I/II or III/IV, based on the Cheng’s classification). In addition, we compared the effectiveness of lenvatinib/sorafenib monotherapy and combination therapy in HCC patients with PVTT, respectively.

### Tumor response and safety assessments

Tumor response was documented by CT or MRI 4 weeks after RT completion. Tumor response was assessed according to the Modified Response Evaluation Criteria in Solid Tumors, including complete response (CR), partial response (PR), stable disease (SD), and progressive disease (PD). AEs occurring within 4 weeks after the end of RT were recorded according to the CTCAE version 5.0. Liver function test, including the measurement of total bilirubin serum level, albumin level, and prothrombin time, were also performed during treatment to assess the toxicity of the treatment to the liver.

### Statistical analysis

All statistical analyses were performed using IBM SPSS Statistics for Windows (version 26.0, IBM Corp., Armonk, N.Y., United States). Independent-sample t-tests and χ^2^ tests were used to compare the differences in baseline characteristics between the two groups. Survival curves were calculated using the Kaplan-Meier method for the two treatment groups. Univariate analysis was performed using the log-rank test. Variables with *p* < 0.10 in the univariate analysis were included in the multivariate analysis. The Cox proportional hazards regression model was used for multivariate analysis. *p* < 0.05 was defined as statistically significant.

## Results

### Patient characteristics

**FIGURE 1:**
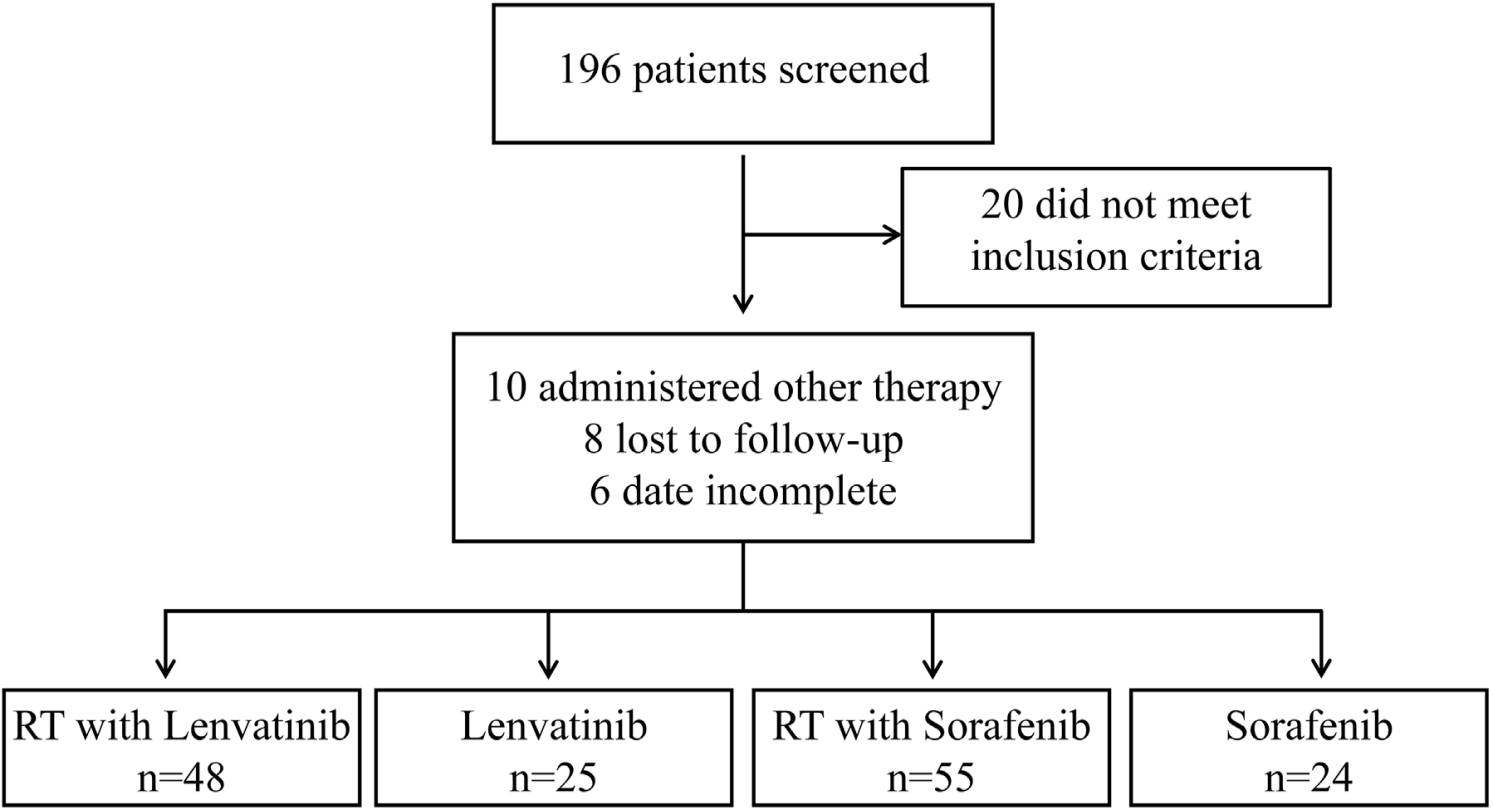
Flow chart for selecting HCC patients with PVTT for this study.

From March 2017 to September 2022, 196 patients with HCC with PVTT received treatment in the Department of Radiotherapy of the First Bethune Hospital of Jilin University. A flow diagram of patient enrollment was shown in Figure 1. 20 patients did not meet the inclusion criteria. 24 patients were excluded because they received other treatments, were lost to follow-up, or had incomplete data. Finally, the study enrolled 152 patients, of which 25 received lenvatinib, 24 received sorafenib, 48 received RT plus lenvatinib and 55 received RT plus sorafenib. The baseline clinicopathologic characteristics of the enrolled patients in each group are shown in Table 1 and Supplementary Table S1. The mean ages of the RT plus lenvatinib and RT plus sorafenib groups were 56.83 ± 8.059 years and 58.67 ± 7.720 years, respectively. The baseline characteristics between patients of the two groups, including sex, ECOG score, PVTT classification, and AFP level, did not differ significantly.

**TABLE 1:** Baseline patient characteristics.

Characteristic	Total (N = 103)	RT + lenvatinib (n = 48)	RT + sorafenib (n = 55)	P
Sex				0.826
Male	76 (73.8%)	36 (75.0%)	40 (72.7%)	
Female	27 (26.2%)	12 (25.0%)	15 (27.3%)	
Age (y)	57.82 ± 7.895	56.83 ± 8.059	58.67 ± 7.720	0.240
ECOG score				0.542
0	38 (36.9%)	16 (33.3%)	22 (40.0%)	
1-2	65 (63.1%)	32 (66.7%)	33 (60.0%)	
Etiology				0.188
HBV	66 (64.1%)	33 (68.8%)	33 (60.0%)	
HCV	14 (13.6%)	8 (16.7%)	6 (10.9%)	
Non-B/C	23 (22.3%)	7 (14.6%)	16 (29.1%)	
Ascites				0.542
Present	38 (36.9%)	16 (33.3%)	22 (40.0%)	
Absent	65 (63.1%)	32 (66.7%)	33 (60.0%)	
AFP level				0.690
>400	44 (42.7%)	22 (45.8%)	22 (40.0%)	
≤400	59 (57.3%)	26 (54.2%)	33 (60.0%)	
Type of PVTT				0.235
I-II	55 (53.4%)	29 (60.4%)	26 (47.3%)	
III-IV	48 (46.6%)	19 (39.6%)	29 (52.7%)	
Lymphatic metastasis				0.398
Absent	70 (68.0%)	35 (72.9%)	35 (63.6%)	
Present	33 (32.0%)	13 (27.1%)	20 (36.4%)	
Tumor stage				0.555
III	52 (50.5%)	26 (54.2%)	26 (47.3%)	
IV	51 (49.5%)	22 (45.8%)	29 (52.7%)	
Number of tumor				0.683
Single	37 (35.9%)	16 (33.3%)	21 (38.2%)	
Multiple	66 (64.1%)	32 (66.7%)	34 (61.8%)	
Child–Pugh				0.420
A	64 (62.1%)	32 (66.7%)	32 (58.2%)	
B	39 (37.9%)	16 (33.3%)	23 (41.8%)	

### Treatment efficacy

**FIGURE 2:**
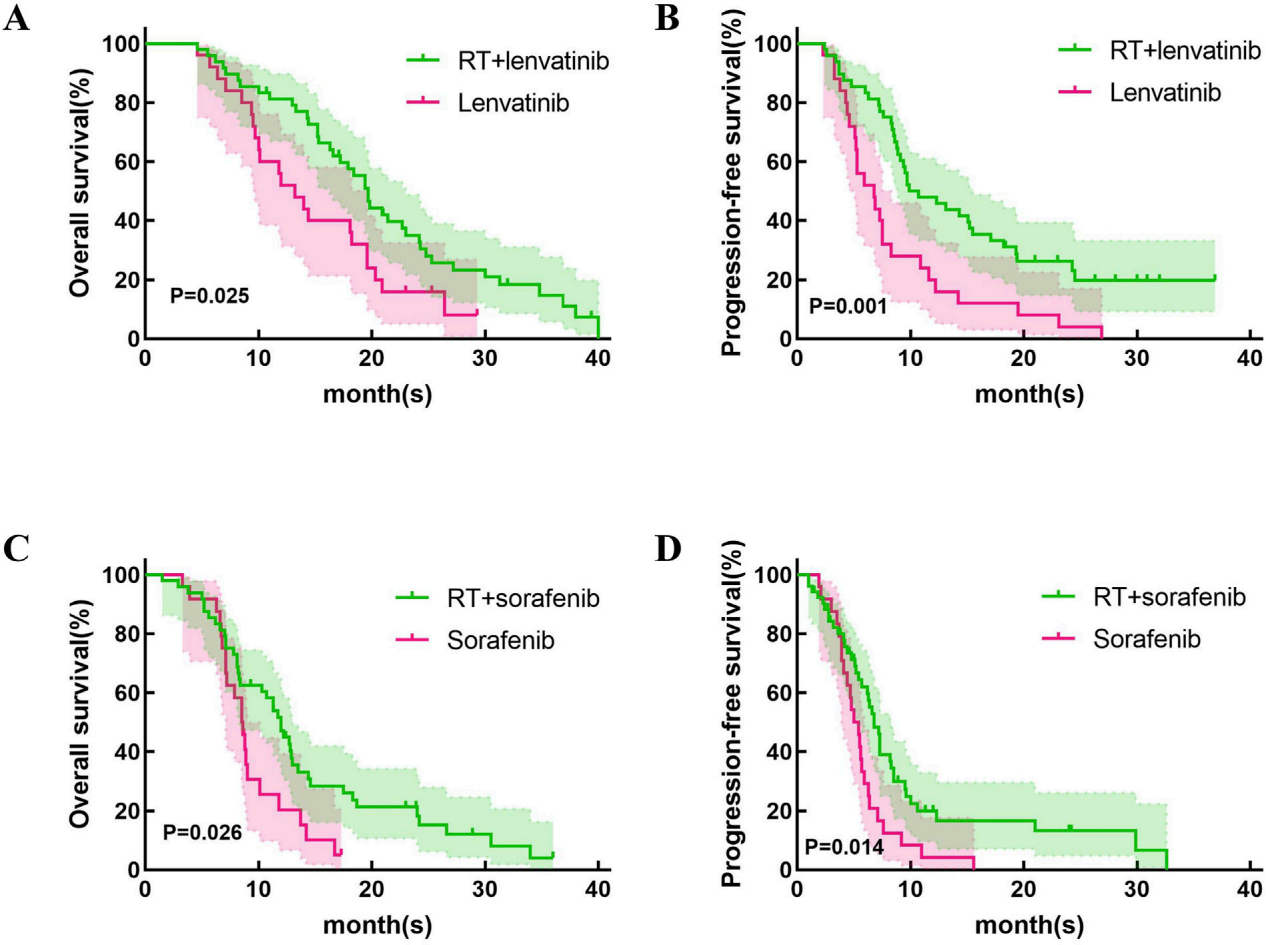
Kaplan-Meier curves of OS and PFS in patients with PVTT who underwent RT + lenvatinib/sorafenib or lenvatinib/sorafenib monotherapy. **(A)**The OS in the patients who underwent RT + lenvatinib or lenvatinib monotherapy. **(B)** The PFS in the patients who underwent RT + lenvatinib or lenvatinib monotherapy. **(C)**The OS in the patients who underwent RT + sorafenib or sorafenib monotherapy. **(D)** The PFS in the patients who underwent RT + sorafenib or sorafenib monotherapy.

**FIGURE 3:**
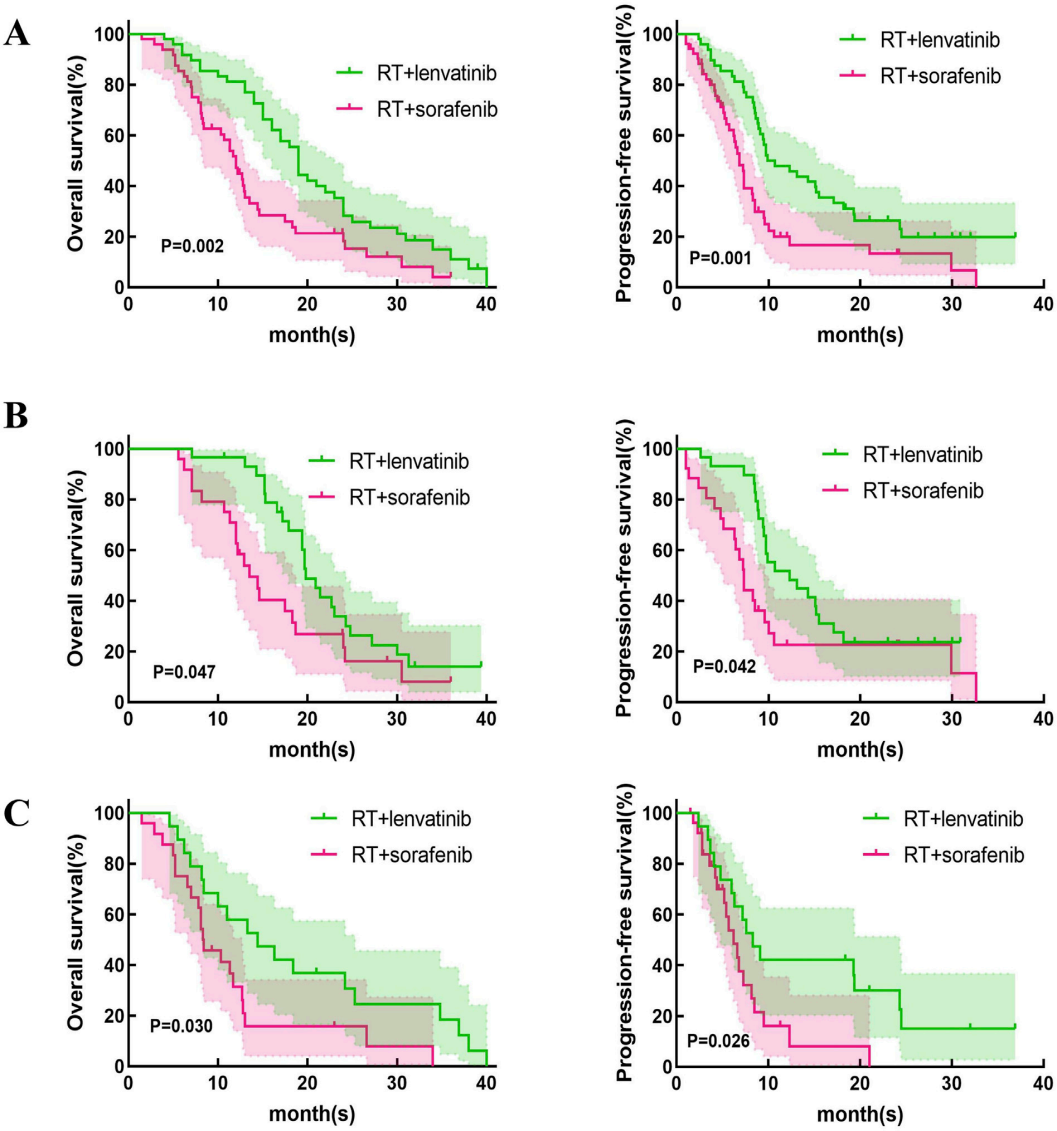
Kaplan-Meier curves of OS and PFS in patients with PVTT who underwent RT + lenvatinib or RT + sorafenib. **(A)** The OS and PFS in the total population. **(B)** The OS and PFS in type I/II PVTT patients. **(C)** The OS and PFS in type III/IV PVTT patients.

This study first compared the efficacy of lenvatinib/sorafenib monotherapy versus RT in HCC patients with PVTT. Our results showed that both median OS and PFS were significantly longer for lenvatinib/sorafenib combined RT than for monotherapy (Figures 2A–D). In addition, the median OS and PFS of patients receiving RT for the entire cohort were 15.2 months and 8.5 months, respectively. The median OS was significantly longer for patients in the RT plus lenvatinib group than for those in the RT plus sorafenib group vs 12.0 months (Figure 3A). Patients treated with RT plus lenvatinib also had a significantly longer median PFS (9.9 vs 6.8 months) (Figure 3A). Additionally, we performed a subgroup analysis of RT plus lenvatinib versus RT plus lenvatinib. For patients with type I/II PVTT, the median OS (19.8 vs 13.5 months) (Figure 3B) and PFS (12.3 vs 7.3 months) (Figure 3B) in those in the RT plus lenvatinib group were longer than for those in the RT plus sorafenib group. For patients with type III/IV PVTT, those in the RT plus lenvatinib group also showed an increased benefit in median OS (14.4 vs 8.3 months) (Figure 3C) and PFS (8.3 vs 6.2 months) (Figure 3C) than those in the RT plus sorafenib group.

### Univariate and multivariate analyses of OS

Univariate analysis performed using log-rank tests to determine the prognostic factors affecting OS showed that etiology, Type of PVTT, AFP level, treatment method, and Child–Pugh score were associated with OS (Table 2). And the factors that affect PFS include treatment method and Child–Pugh score. Multivariate analyses performed based on Cox regression models to identify independent prognostic factors associated with PFS showed that AFP level, treatment method, Child–Pugh score and Number of tumor were identified as independent prognostic factors for PFS (Table 3). And the independent prognostic factors that affect PFS include treatment method, etiology, AFP level, and Child–Pugh (Table 4).

**TABLE 2:** Univariate analysis of prognostic factor for PFS and OS.

Factor	Total (N)	Median PFS (m)	P	Median OS (m)	P
Sex			0.470		0.936
Male	76	8.5		18.3	
Female	27	8.8		14.4	
Age			0.597		0.971
>60	38	9.5		15.2	
≤60	65	8.5		14.4	
Etiology			0.158		0.002
HBV/HCV	80	8.8		17.9	
Non-HBV/HCV	23	6.8		8.3	
Type of PVTT			0.113		0.034
I-II	55	9.6		18.7	
III-IV	48	6.8		11.0	
Tumor stage			0.518		0.707
III	52	9.4		16.6	
IV	51	8.2		14.6	
Number of tumor			0.077		0.671
Single	37	7.3		17.5	
Multiple	66	9.4		14.4	
AFP level			0.060		0.003
>400	44	8.3		12.0	
≤400	59	9.1		18.7	
Treatment method			0.001		0.002
RT + lenvatinib	48	9.9		19.7	
RT + sorafenib	55	6.8		12.0	
Child-Pugh			<0.001		<0.001
A	64	9.9		19.7	
B	39	6.2		8.2	
Ascites			0.726		0.612
Present	38	8.9		14.3	
Absent	65	8.5		15.2	
ECOG score			0.823		0.371
0	38	7.3		14.3	
1-2	65	8.8		15.2	

**TABLE 3:** Multivariate analysis of prognostic factors for PFS.

Factor	HR	P
Treatment method		
RT + lenvatinib	1	0.008
RT + sorafenib	1.841 (1.172-2.893)	
AFP level		
>400	1	0.024
≤400	0.574 (0.355-0.929)	
Child-Pugh		
A	1	<0.001
B	2.614 (1.600-4.269)	
Number of tumor		0.013
Single	1	
Multiple	0.547 (0.339-0.881)	

**TABLE 4:** Multivariate analysis of prognostic factors for OS.

Factor	HR	P
Treatment method		
RT + lenvatinib	1	0.002
RT + sorafenib	2.023 (1.288-3.180)	
Etiology		
Non-HBV/HCV	1	0.036
HBV/HCV	0.532 (0.294-0.961)	
AFP level		
>400	1	0.005
≤400	0.511 (0.318-0.821)	
Child-Pugh		
A	1	<0.001
B	3.151 (1.977-5.021)	

### Tumor response

The tumor responses for patients in the RT plus lenvatinib and RT plus sorafenib groups are shown in Table 5. According to the CR, PR, SD, and PD criteria, no patients in either group had a CR. In the RT plus lenvatinib group, 25.0% (n = 12) achieved a PR, 50.0% (n = 24) achieved SD, and 25.0% (n = 12) showed PD. In contrast, 20.0% (n = 11) of the patients in the RT plus sorafenib group achieved a PR, 50.9% (n = 28) achieved SD, and 29.1% (n = 16) showed PD. The ORR in the RT plus lenvatinib group was 25.0%, which was higher than that in the RT plus sorafenib group (20.0%). In addition, the DCRs were 75.0% and 70.9% in the RT plus lenvatinib and RT plus sorafenib groups, respectively.

**TABLE 5:** The tumor response assessed using mRECIST criteria.

Tumor response	RT + lenvatinib	RT + sorafenib
Complete response	0	0
Partial response	12	11
Stable disease	24	28
Progressive disease	12	16
Total patients	48	55
Objective response rate	25.0%	20.0%
Disease control rate	75.0%	70.9%

### Safety outcomes

As shown in Table 6, the safety analysis included 103 patients. Most AEs were grades 1–2. The overall incidence rates of treatment-related AEs were similar between patients of the RT plus lenvatinib and RT plus sorafenib groups. The most frequent AEs in patients of the RT plus lenvatinib group were increased aspartate aminotransferase level (n = 28, 58.3%), increased alanine aminotransferase level (n = 21, 43.8%), increased bilirubin level (n = 22, 45.8%), decreased white blood cell (WBC) count (n = 27, 56.3%), decreased platelet count (n = 31, 64.6%), anemia (n = 15, 31.3%), fatigue (n = 23, 47.9%), diarrhea (n = 7, 14.6%), nausea (n = 18, 37.5%), and vomiting (n = 9, 18.8%). The most common AEs in the RT plus sorafenib group were decreased WBC count (n = 33, 60.0%), decreased platelet count (n = 23, 41.8%), anemia (n = 28, 50.9%), increased aspartate aminotransferase (n = 32, 58.2%), increased alanine aminotransferase (n = 27, 49.1%), increased bilirubin (n = 24, 43.6%), fatigue (n = 20, 36.4%), nausea (n = 22, 40%), vomiting (n = 10, 18.2%), and diarrhea (n = 10, 18.2%). One case of upper gastrointestinal bleeding and one case of abdominal infection occurred in the RT lenvatinib group, respectively, which led to treatment interruption. Two cases of severe diarrhea and pyrexia occurred in the RT plus sorafenib group, respectively, leading to treatment interruption. No treatment-related deaths were observed in either group.

**TABLE 6:** Adverse events.

Adverse events	RT + Lenvatinib (n = 48)			RT + Sorafenib (n = 55)		
	Total n (%)	Grad 1/2	Grade 3/4	Total n (%)	Grade 1/2	Grade 3/4
Elevated AST	28 (58.3)	26	2	32 (58.2)	29	3
Elevated ALT	21 (43.8)	18	3	27 (49.1)	24	3
Increased bilirubin	22 (45.8)	21	1	24 (43.6)	22	2
Decreased WBC	27 (56.3)	25	2	33 (60.0)	27	6
Anemia	15 (31.3)	12	3	28 (50.9)	25	3
Decreased PLT	31 (64.6)	29	2	23 (41.8)	13	10
Hand-foot skin reaction	6 (12.5)	6	0	3 (5.5)	3	0
Fever	3 (6.3)	3	0	5 (9.1)	5	2
Diarrhea	7 (14.6)	6	1	10 (18.2)	8	2
Fatigue	23 (47.9)	18	5	20 (36.4)	15	5
Bleeding	2 (4.2)	2	1	4 (7.3)	4	0
Nausea	18 (37.5)	15	3	22 (40.0)	20	2
Vomiting	9 (18.8)	9	0	10 (18.2)	8	2

## Discussion

PVTT is common in HCC patients and usually shows a poor prognosis. Although PVTT is a common adverse prognostic factor for HCC, no standard treatment regimen is available. Chen et al. were the first to demonstrate the efficacy of RT for PVTT. All 10 patients with PVTT included in their study received irradiation doses of 30–50 Gy, among which five patients had portal vein invasion that completely disappeared and the other five patients had partial contraction (Chen et al., 1994). Tang et al. have reported that among 371 patients with resectable HCC and PVTT, the median OS was 2.3 months longer in patients receiving RT than in those receiving surgical resection (Tang et al., 2013). PVTT has been identified as one of the indications for RT in patients with HCC. However, there is still no uniform standard on how to combine local treatment with systemic treatment.

Lenvatinib and sorafenib are both multi-kinase inhibitors and are recommended as first-line therapies for advanced HCC (Sun et al., 2022). We first explored the difference in efficacy between RT combined with lenvatinib/sorafenib versus monotherapy. Similar to previous studies (Yu et al., 2022; Rim et al., 2021), RT combined with sorafenib is significantly more effective than sorafenib monotherapy in HCC patients with PVTT. In addition, our study showed that patients treated with RT plus lenvatinib had longer OS and PFS than those treated with lenvatinib monotherapy (*p* = 0.025 and *p* = 0.001).

However, which of lenvatinib or sorafenib is more suitable for use in combination with RT in HCC patients with PVTT? In the REFLECT trial, compared to sorafenib, lenvatinib monotherapy showed significantly superior PFS (7.2 vs 4.6 months) and ORR (29.6% vs 6.9%) for patients with unresectable HCC (Yamashita et al., 2020). A recent meta-analysis compared efficacy and safety between lenvatinib and sorafenib in HCC treatment (Hua et al., 2024). The study found that treatment with lenvatinib in HCC patients resulted in better OS, PFS, and higher ORR and DCR compared to sorafenib. In the treatment of HCC with PVTT, combination therapy provides a higher OS than sorafenib or lenvatinib alone (Ding et al., 2021). Our study compared the efficacy and safety of RT plus lenvatinib and RT plus sorafenib in patients with HCC and PVTT. Our results showed that the median OS of the patients treated by RT plus lenvatinib and sorafenib is 19.7 and 12.0 months (*p* = 0.024). Our results showed that RT plus lenvatinib had a significant survival benefit. The results may come from the spatial cooperation between RT targeting macroscopic disease and lenvatinib controlling the underlying microscopic disease. Lenvatinib targets VEGF receptors one to three, FGF receptors one to four, PDGF receptor-a, RET, and KIT proto-oncogene products, and enhances oxygen effects by normalizing the vasculature of surviving tumors (Matsui et al., 2008a; Matsui et al., 2008b). External beam RT induces mitotic death through DNA damage and immunogenic stimulation (Lee et al., 2020). The combination of these two treatment modalities may be reasonable. In addition, our results revealed that patients receiving RT plus lenvatinib also showed better ORR and DCR than those receiving RT plus sorafenib. We divided these patients into two subgroups based on PVTT classification (PVTT-I/II and PVTT-III/IV), and the analysis results suggested that in both PVTT-I/II and PVTT-III/IV groups, patients treated with RT plus lenvatinib had longer median OS and PFS than RT plus sorafenib.

In univariate analysis, there was no significant difference in median OS between patients with different sex, ages, tumor stage and number of tumors. For HCC patients with PVTT, hepatitis virus infection, PVTT typing, AFP level, treatment method, and Child-Pugh showed significant effects on OS. Multivariate analyses performed based on Cox regression models to identify independent prognostic factors associated with OS. The influencing factors with *p* < 0.10 after univariate analysis were included in the multivariate analysis. In this retrospective study, treatment method, etiology, AFP level and Child-Pugh were identified as independent prognostic factors for OS.

In this study, the safety profile of lenvatinib plus RT was similar to that of sorafenib plus RT. The most frequent AEs were increased aspartate aminotransferase level (n = 28, 58.3%), increased alanine aminotransferase level (n = 21, 43.8%), increased bilirubin level (n = 22, 45.8%), decreased white blood cell (WBC) count (n = 27, 56.3%), decreased platelet count (n = 31, 64.6%), anemia (n = 15, 31.3%), fatigue (n = 23, 47.9%), diarrhea (n = 7, 14.6%), nausea (n = 18, 37.5%), and vomiting (n = 9, 18.8%). These findings are consistent with those reported previously. These AEs can be relieved by dose adjustments or symptomatic treatment. No treatment-related deaths were observed in our study. Most AEs were grade 1 or 2. These results suggested that RT plus lenvatinib is safe in patients with PVTT.

This study had several limitations. First, it was a retrospective study, which led to selection bias in the study population. The treatment options for patients with PVTT were determined according to the preferences of the attending physician, and some patients received TACE before RT at different times. Second, our study had a small sample size, which reduced its statistical power. The small sample size may have led to deviations in the median OS and PFS. Therefore, prospective studies with larger sample sizes are needed for further confirmation. In conclusion, the results of this study showed that RT plus lenvatinib had a significant advantage over RT + sorafenib in terms of OS and PFS in patients with PVTT. RT plus lenvatinib was also safe and tolerable in patients with PVTT. Therefore, RT plus lenvatinib is an effective treatment option for patients with PVTT. However, the timing and sequence of the combination of RT and systemic therapy remain controversial.

## Data Availability

The raw data supporting the conclusions of this article will be made available by the authors, without undue reservation.

## Glossary

HCC: hepatocellular carcinoma
PVTT: portal vein tumor thrombus
NCCN: National Comprehensive Cancer Network
OS: overall survival
TACE: transcatheter arterial chemoembolization
RT: radiotherapy
VEGF: vascular endothelial growth factor
FGF: fibroblast growth factor
PDGF: platelet-derived growth factor
PFS: progression-free survival
ORR: objective response rate
HRQOL: health-related quality of life
AFP: alpha-fetoprotein
TTP: time to progression
3D-CRT: three-dimensional conformal radiotherapy
IMRT: intensity-modulated radiotherapy
SBRT: stereotactic body radiotherapy
IC: intrahepatic control
LC: local control
MRI: magnetic resonance imaging
CT: computer tomography
ECOG: Eastern Cooperative Oncology Group
GTV: gross tumor volume
CTV: clinical tumor volume
PTV: planning target volume
AE: adverse event
CTCAE: Common Terminology Criteria for Adverse Events
WBC: white blood cell
CI: confidence interval
HR: hazard ratio
CR: complete response
PR: partial response
SD: stable disease
PD: progressive disease.
