# Supplementary material for: Radiotherapy plus lenvatinib versus radiotherapy plus sorafenib for hepatocellular carcinoma with portal vein tumor thrombus: a retrospective study
Source: Front Pharmacol. 2024 Oct 15;15:1458819. doi: 10.3389/fphar.2024.1458819 (PMC11518791; doi:10.3389/fphar.2024.1458819)
Supplement: Supplementary file 1 [file Table1.docx]

Table S1. Baseline Patient Characteristics

| Characteristic | Lenvatinib  n (%) | RT + lenvatinib  n (%) | P value | Sorafenib  n (%) | RT + sorafenib  n (%) | P value |
| --- | --- | --- | --- | --- | --- | --- |
| Sex |  |  | 0.785 |  |  | 1.000 |
| Male | 18（72.0） | 36（75.0） |  | 18（75.0） | 40（72.7） |  |
| Female | 7（28.0） | 12（25.0） |  | 6（25.0） | 15（27.3） |  |
| Age (y) | 56.52 ± 7.484 | 56.83 ± 8.059 | 0.872 | 58.92 ± 7.089 | 58.67 ± 7.720 | 0.895 |
| ECOG score |  |  | 0.138 |  |  | 1.000 |
| 0 | 13（52.0） | 16（33.3） |  | 9（37.5） | 22（40.0） |  |
| 1-2 | 12（48.0） | 32（66.7） |  | 15（62.5） | 33（60.0） |  |
| Etiology |  |  | 0.366 |  |  | 0.478 |
| HBV | 13（52.0） | 33（68.8） |  | 12（50.0） | 33（60.0） |  |
| HCV | 6（24.0） | 8（16.7） |  | 5（20.8） | 6（10.9） |  |
| Non-B/C | 6（24.0） | 7（14.6） |  | 7（29.2） | 16（29.1） |  |
| Ascites |  |  | 0.794 |  |  | 0.623 |
| Present | 7（28.0） | 16（33.3） |  | 8（33.3） | 22（40.0） |  |
| Absent | 18（72.0） | 32（66.7） |  | 16（66.7） | 33（60.0） |  |
| AFP level |  |  | 0.208 |  |  | 0.623 |
| >400 | 7（39.7） | 22（45.8） |  | 8（33.3） | 22（40.0） |  |
| ≤400 | 18（60.3） | 26（54.2） |  | 16（66.7） | 33（60.0） |  |
| Type of PVTT |  |  | 0.619 |  |  | 1.000 |
| I-II | 13（52.0） | 29（60.4） |  | 12（50.0） | 26（47.3） |  |
| III-IV | 12（48.0） | 19（39.6） |  | 12（50.0） | 29（52.7） |  |
| Lymphatic metastasis |  |  | 0.296 |  |  | 0.321 |
| Absent | 15（60.0） | 35（72.9） |  | 12（50.0） | 35（63.6） |  |
| Present | 10（40.0） | 13（27.1） |  | 12（50.0） | 20（36.4） |  |
| Tumor stage |  |  | 1.000 |  |  | 0.325 |
| III | 14（56.0） | 26（54.2） |  | 8（33.3） | 26（47.3） |  |
| IV | 11（44.0） | 22（45.8） |  | 16（66.7） | 29（52.7） |  |
| Number of tumor |  |  | 1.000 |  |  | 0.802 |
| Single | 8（32.0） | 16（33.3） |  | 8（33.3） | 21（38.2） |  |
| Multiple | 17（68.0） | 32（66.7） |  | 16（66.7） | 34（61.8） |  |
| Child–Pugh |  |  | 0.792 |  |  | 0.807 |
| A | 18（72.0） | 32（66.7） |  | 13（54.2） | 32（58.2） |  |
| B | 7（28.0） | 16（33.3） |  | 11（45.8） | 23（41.8） |  |
